# Sports promote brain evolution: a resting-state fMRI study of volleyball athlete

**DOI:** 10.3389/fspor.2024.1393988

**Published:** 2024-05-02

**Authors:** Jun-Peng Zhang, Ping Zhu, Zeng-Liang Cai, Xiang-Xin Xing, Jia-Jia Wu, Mou-Xiong Zheng, Xu-Yun Hua, Bo-Min Gong, Jian-Guang Xu

**Affiliations:** ^1^ School of Rehabilitation Science, Shanghai University of Traditional Chinese Medicine, Shanghai, China; ^2^ Engineering Research Center of Traditional Chinese Medicine Intelligent Rehabilitation, Ministry of Education, Shanghai, China; ^3^ Department of Physical Education, Shanghai University of Traditional Chinese Medicine, Shanghai, China; ^4^ Department of Rehabilitation Medicine, Yueyang Hospital of Integrated Traditional Chinese and Western Medicine, Shanghai University of Traditional Chinese Medicine, Shanghai, China; ^5^ Department of Traumatology and Orthopedics, Yueyang Hospital of Integrated Traditional Chinese and Western Medicine, Shanghai University of Traditional Chinese Medicine, Shanghai, China

**Keywords:** volleyball, functional connection, athlete, brain plasticity, fMRI

## Abstract

**Background:**

Long-term skill learning can lead to structure and function changes in the brain. Different sports can trigger neuroplasticity in distinct brain regions. Volleyball, as one of the most popular team sports, heavily relies on individual abilities such as perception and prediction for high-level athletes to excel. However, the specific brain mechanisms that contribute to the superior performance of volleyball athletes compared to non-athletes remain unclear.

**Method:**

We conducted a study involving the recruitment of ten female volleyball athletes and ten regular female college students, forming the athlete and novice groups, respectively. Comprehensive behavioral assessments, including Functional Movement Screen and audio-visual reaction time tests, were administered to both groups. Additionally, resting-state magnetic resonance imaging (MRI) data were acquired for both groups. Subsequently, we conducted in-depth analyses, focusing on the amplitude of low-frequency fluctuations (ALFF), regional homogeneity (ReHo), and functional connectivity (FC) in the brain for both the athlete and novice groups.

**Results:**

No significant differences were observed in the behavioral data between the two groups. However, the athlete group exhibited noteworthy enhancements in both the ALFF and ReHo within the visual cortex compared to the novice group. Moreover, the functional connectivity between the visual cortex and key brain regions, including the left primary sensory cortex, left supplementary motor cortex, right insula, left superior temporal gyrus, and left inferior parietal lobule, was notably stronger in the athlete group than in the novice group.

**Conclusion:**

This study has unveiled the remarkable impact of volleyball athletes on various brain functions related to vision, movement, and cognition. It indicates that volleyball, as a team-based competitive activity, fosters the advancement of visual, cognitive, and motor skills. These findings lend additional support to the early cultivation of sports talents and the comprehensive development of adolescents. Furthermore, they offer fresh perspectives on preventing and treating movement-related disorders.

**Trial registration:**

Registration number: ChiCTR2400079602. Date of Registration: January 8, 2024.

## 1 Introduction

Over time, scholars have discovered through research on human movement and the brain that long-term acquisition of motor skills can lead to plastic reorganization of brain structure and function, resulting in improved perceptual, cognitive, and motor abilities (1, 2). When comparing the performance of athletes at different levels, experts demonstrate exceptional skills in specific tasks related to their respective sports compared to sub-elite athletes and novice individuals. These superior abilities include the utilization of advanced visual cues (3), effective visual search strategies (4), precise prediction, and decision-making skills (5). Furthermore, sustained engagement in physical activity brings about changes in the prefrontal cortex and temporal lobe, while also leading to increased hippocampal volume, thereby enhancing cognitive function and fostering brain plasticity (4). Such plasticity is evident in elite athletes who have undergone extensive training in sports such as golf (6), badminton (7), football (8), gymnastics (1), and swimming (9).

Indeed, in various sports, different brain regions undergo resting-state neuroplastic changes (10). These brain regions are intricately involved in motor judgment, perception, prediction, execution, and long-term specific task training. Consequently, athletes gain the ability to make rapid and precise judgments and responses to both internal and external stimuli in specific situations (11).

Volleyball is one of the most widely played sports, with millions of participants worldwide (12). It is a comprehensive and competitive team sport that requires athletes to possess technical skills, tactics, and good physical fitness. They must be able to perform repetitive jumping, multidirectional movements, and sustain prolonged matches (13). The ability to anticipate events and actions in volleyball is crucial for excellent performance. This necessitates better recognition and storage of complex movement patterns and the ability to predict and perceive the current environment (4). Scholars have found that as the level of volleyball teams improves, there is less variation among individual players within the team. Factors such as decision-making abilities, performance under pressure, tactical skills, and knowledge become decisive in distinguishing players' performance (14).

So how do these factors affect athletes' performance levels? Research has shown that volleyball players perform better in tasks involving motor control and visual-spatial attention processing (15). Quick motor reactions in sports often require the ability to anticipate the opponent's anticipated actions early. Athletes look for relevant movement patterns based on cues from other players' body postures, such as elbow angles (16). Researchers attribute athletes' superior performance to the central nervous system, where the action observation network plays a crucial role in the motion prediction process for professional volleyball players (16, 17). Specifically, the posterior cingulate cortex and supplementary motor area have been found to correlate positively with anticipation abilities, indicating that athletes, through long-term professional training, develop better perceptual-motor representations in that domain (18). However, current research on volleyball players primarily focuses on training cycles, multidirectional movement abilities, and physical attributes, lacking in-depth exploration of the brain mechanisms underlying the advanced abilities required by elite players, such as perception, judgment, and decision-making skills (13).

Exercise not only benefits athletes by promoting brain spatial memory and other functions, but it has also been shown in other studies to reduce the risk of cognitive disorders, cardiovascular diseases, diabetes, and obesity (19). To explore the mechanisms of exercise on brain plasticity, simple exercise mechanisms can be precisely located and studied using animal models to observe changes in brain circuits. However, for humans participating in more complex tasks, such as team sports like volleyball, it is challenging to explore relevant brain mechanisms through basic animal experiments. By utilizing functional magnetic resonance imaging (fMRI), we can accurately locate brain regions and observe changes in interregional connections, thus exploring the plasticity changes in the human brain caused by complex team sports activities.

In neuroscience and cognitive science, fMRI is widely used. It allows researchers to non-invasively observe brain activity by measuring changes in blood flow and oxygenation in response to various tasks or stimuli (20, 21). fMRI is employed to assess changes in brain activation in both cortical and subcortical regions. Its high spatial resolution enables precise localization of distinct brain areas and facilitates the investigation of functional and structural differences in diverse populations. Additionally, Electroencephalogram and functional near-infrared spectroscopy are also used to explore changes in neural efficiency among athletes (22). The advancements in neuroimaging have provided new insights into the functional reorganization associated with the acquisition, consolidation, and retention of motor skills (5).

This study aims to use fMRI to measure the activity levels of brain regions during the resting state and investigate functional connectivity changes in differential brain areas, visualizing characteristic brain regions in female college volleyball players. By combining the physical fitness test scores and results of audiovisual reaction time, the study analyzes the differences in motor cognition between female college volleyball players and regular college students, exploring the neuroplastic changes induced by volleyball.

## 2 Methods

### 2.1 Participants

This study recruited ten national level 2 or above women's volleyball players as the athlete group from Shanghai University of Traditional Chinese Medicine, and ten students inexperienced in volleyball as the novice group. The athlete group consisted of ten female participants, aged between 19 and 23 years (average age 21.0 ± 1.3 years), with 8–12 years of volleyball training (average period 10.3 ± 1.5 years). The novice group consisted of ten subjects, aged between 20 and 22 years (average age 20.4 ± 0.7), with 0.5–1 years of tai chi chuan training (average period 0.7 ± 0.21 years). The inclusion criteria for all subjects were: (1) visual acuity of at least level 1.0 or corrected visual acuity, (2) Female participants aged 18–30 years old were included, (3) right-handedness, (4) meeting the MRI scan conditions, such as having no metal inserts in the body and no phobia of sealing; and (5) no history of brain trauma or neurological or mental illness. The exclusion criteria for all subjects were (1) Taking medications that alter cortical excitability in the brain (such as antiepileptic drugs, sedatives, or hypnotics) within the past three months, (2) Having auditory or visual impairments that may affect the assessment. Further details are presented in Table 1. To avoid assessment bias, each item in the behavioral assessments was completed by a single researcher within one week.

**Table 1:** Basic information.

Parameter	Athletes	Novices
	Mean ± SD	Mean ± SD
Age	21.00 ± 1.30^a^	20.40 ± 0.70
Years of sports experience	10.30 ± 1.50	0.70 ± 0.21
FMS	16.8 ± 1.87^a^	17.50 ± 1.08
Auditory reaction time	174.22 ± 32.49^a^	184.04 ± 23.75
Visual reaction time	622.42 ± 95.02^a^	591.85 ± 57.14
SDMT	69.5 ± 7.83^a^	68.80 ± 9.28
HAM-A	5.40 ± 4.12^a^	7.00 ± 4.64
HAMD	6.50 ± 5.08^a^	8.80 ± 5.65

FMS, functional movement screen; SDMT, the symbol digit modalities test; HAM-A, the hamilton rating scale for anxiety; HAMD, hamilton depression scale.

^a^ No significantly different to novices (*p* > 0.05).

### 2.2 Functional movement screen

The Functional Movement Screen (FMS) test consists of seven components: deep squat, hurdle step, inline lunge, shoulder mobility, straight leg raise, trunk stability push-up, and rotary stability (23). Each component is scored on a scale of 3, 2, 1, or 0. A score of 3 indicates that the subject can perform the required movement without any compensation or instability. A score of 2 indicates that the subject can complete the movement but with some instability or compensation, or by performing a modified version of the movement. A score of 1 indicates that the subject is unable to perform the movement as required. However, it is important to note that all scoring is based on the absence of pain during the movement or when there is no pain during the exclusionary testing. If pain is present, the movement is scored as 0.

### 2.3 Measurement of audio-visual reaction time

The EP 204 audio-visual reaction time measuring instrument (produced by the Science and Education Instrument Factory of East China Normal University) was used in this test (24). The instrument automatically presents auditory and visual (four-color light simultaneously) stimuli. In each experiment, the number of presentations of light and sound stimuli is equal, and if the test is set to 20 times, then each will be presented 10 times in a random order. When the subject hears (or sees) the stimuli, they must lift their finger from the circular hole on the response keyboard. The instrument automatically records the time from the presentation of the stimulus to the subject lifting their finger from the circular hole on the response keyboard, which is the reaction time for the sound (or light). During the experiment, if the subject does not put their finger back into the circular hole on the response keyboard, the instrument will automatically enter a waiting state until the subject puts their finger back and the next test can be performed.

### 2.4 Symbol digit modalities test

The Symbol Digit Modalities Test (SDMT) consists of 9 pairs of alphanumeric symbols (25). The participants are required to fill in the appropriate symbol below each number within a specified time limit, based on the given alphanumeric symbol relationship. This test primarily measures attention, the endurance of simple sensory-motor processes, the ability to establish new associations, and speed.

### 2.5 Hamilton rating scale for anxiety

The Hamilton Rating Scale for Anxiety (HAM-A), developed by Hamilton in 1959, is a commonly used scale in clinical psychiatry (26). It consists of 14 items. Each item on the HAM-A is scored on a 5-point rating scale ranging from 0 to 4, with the following criteria: (0) None, (1) Mild, (2) Moderate, (3) Severe, and (4) Very Severe. The total score reflects the severity of the condition. According to data provided by the National Scale Collaboration Group, a total score exceeding 29 indicates severe anxiety, exceeding 21 indicates definite anxiety, exceeding 14 indicates the presence of anxiety, and exceeding 7 suggests possible anxiety. A score below 6 indicates the absence of anxiety symptoms. The commonly used cutoff point for the 14-item HAM-A is 14.

### 2.6 Hamilton depression scale

The 24-item Hamilton Depression Scale (HAMD), developed by Hamilton, is widely used in clinical settings to assess depressive states (27). It is applicable to adults experiencing depressive symptoms. Most items on the HAMD utilize a 5-point rating scale ranging from 0 to 4. The criteria for each level are as follows: (0) None, (1) Mild, (2) Moderate, (3) Severe, and (4) Very Severe. A few items use a 3-point rating scale ranging from 0 to 2, with the following criteria: (0) None, (1) Mild to Moderate, and (2) Severe. The interpretation of the total score is as follows: a score of <8 indicates normal, a score between 8 and 20 suggests possible depression, a score between 20 and 35 indicates definite depression, and a score >35 signifies severe depression.

### 2.7 fMRI acquisition

All data acquisition was performed using the Siemens MAGNETOM Verio 3.0 T magnetic resonance imaging system in Germany. To ensure that the data was not influenced by time, all participants' fMRI scans were collected within one week, primarily in the afternoons of each day. When conducting fMRI scans, we provide subjects with instructions regarding the scanning process. These instructions typically include basic precautions such as avoiding metallic implants and refraining from wearing metal objects during the scan. Additionally, participants are informed to remain still and relaxed during resting-state MRI scans to ensure the integrity of the data. We also employ video monitoring to observe the subjects' status during the data acquisition process.

The Echo Planar Imaging (EPI) scan parameters were: repetition time (TR) = 3,000 ms; echo time (TE) = 30 ms; flip angle = 90°; slice thickness = 3 mm; number of slices = 43; matrix size = 64 × 64; field of view (FOV) = 230 mm × 230 mm; voxel size = 3.6 mm × 3.6 mm × 3 mm; and a total of 200 time points were acquired. The T1 sequence scan parameters were: TR = 1,900 ms; inversion time = 900 ms; TE = 2.93 ms; flip angle = 9°; FOV = 256 mm × 256 mm; slice thickness = 1 mm; matrix size = 256 × 256.

### 2.8 Preprocessing

The data were preprocessed using Restplus 1.25 based on SPM12 (28). The preprocessing steps included removing the first ten time points, performing slice timing correction, realignment, reorientation, normalization, and smoothing (with a full-width at half-maximum of 6 mm × 6 mm × 6 mm). Additionally, detrending, nuisance covariate regression, and filtering were applied. Following these preprocessing steps, subsequent metrics were calculated.

### 2.9 Amplitude of low-frequency fluctuation

After preprocessing, Restplus 1.25 was used to calculate the amplitude of low-frequency fluctuations (ALFF) for each voxel at the low-frequency range (usually 0.01–0.08 Hz), reflecting the strength of local voxel activity during resting state (29). The mean ALFF was calculated to obtain mALFF. The ALFF was then converted to zALFF. Comparing the low-frequency (0.01–0.08 Hz) power spectrum of unfiltered data to the power spectrum across the whole frequency range, a fractional ALFF (fALFF) was obtained. fALFF improves the sensitivity and specificity for detecting spontaneous brain activity.

### 2.10 Regional homogeneity

After preprocessing, Restplus 1.25 was used to calculate the Regional Homogeneity (ReHo) using Kendall's coefficient of concordance to measure the synchronization of brain function activity in local regions across the whole brain (30). Coherence-based ReHo (CoHeReho) was further calculated to represent the consistency of signals in the frequency domain, which can help address the impact of phase inconsistency (31).

### 2.11 Functional connection

Based on the results of ALFF and ReHo, significant changes in the regions of interest (ROIs) were identified as seed points. Restplus 1.25 was used to calculate functional connectivity(FC) changes between the ROIs and all voxels in the brain (32).

### 2.12 Statistical analysis

Using SPSS 25.0 software to analyze whether there is a statistical difference in behavior between athletes and ordinary college students. The normality of the raw data was tested, and for data that met normality, a two-sample *t*-test was used. For data that did not meet normality, a two-sample rank-sum test was used. Using Restplus1.25, a two-sample *t*-test was performed on ALFF, ReHo, and FC for the two groups, with alphasim correction. Pearson correlation test was performed on behavioral and imaging data.

## 3 Results

### 3.1 Behavior

There were no significant differences observed between the athlete group and the novice group in terms of FMS, audio-visual reaction time, SDMT, HAM-A, and HAMD (Table 1).

### 3.2 Amplitude of low-frequency fluctuation

**Figure 1:**
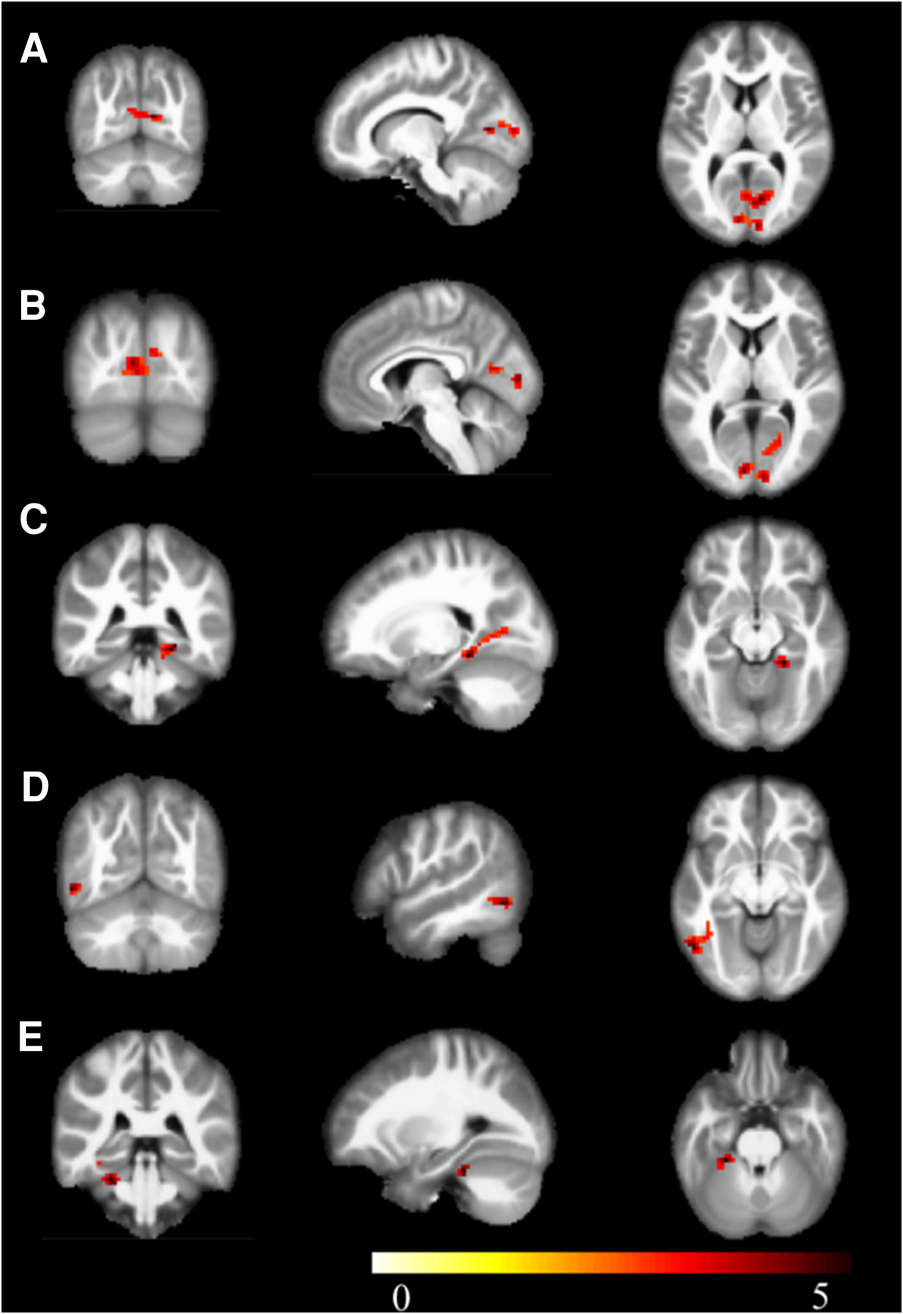
mALFF, warm colors indicate increased activation in the athlete group compared to the novice group. (**A**) Calcarine_R; (**B**) Calcarine_L; (**C**) ParaHippocampal_R; (**D**) Temporal_Inf_L; (**E**) Cerebelum_4_5_L; Cluster *p* < 0.05.

**Figure 2:**
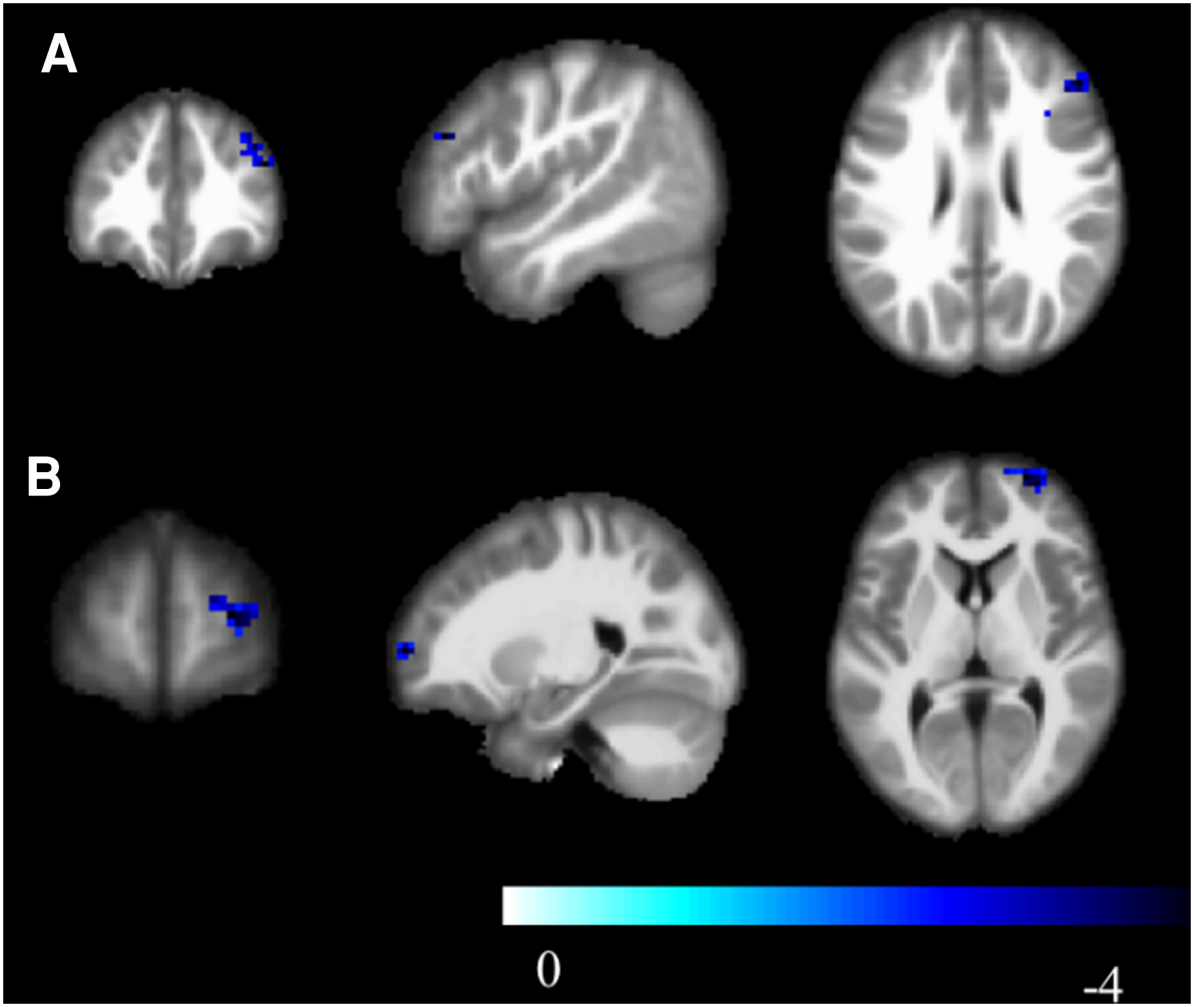
zfALFF, cool colors indicate decreased activation in the athlete group compared to the novice group. (**A**) Frontal_Mid_2_R; (**B**) Frontal_Sup_2_R; Cluster *p* < 0.05.

Compared to the novice group, the athlete group showed significant increases in mALFF in right parahippocampal gyrus, right calcarine cortex, left calcarine cortex, left inferior temporal gyrus, and left lobules 4–5 of the cerebellum (*p* < 0.005) (Figure 1). The zALFF values of right calcarine cortex, left calcarine cortex, right parahippocampal gyrus, and left inferior temporal gyrus, were significantly increased in the athlete group (*p* < 0.005) (Table 2). The mfALFF of right middle frontal gyrus was significantly decreased (*p* < 0.005). The zfALFF of right superior frontal gyrus and right middle frontal gyrus were significantly decreased (*p* < 0.005) (Figure 2, Table 3).

**Table 2:** Brain regions with heightened activity in athletes compared to novices.

Positive region label	Cluster extent	*t*-value	MNI coordinates		
			x	y	z
mALFF					
ParaHippocampal_R	221	5.021	21	−39	−12
Calcarine_R	221	4.522	12	−72	9
Calcarine_L	221	4.099	−6	−87	6
Temporal_Inf_L	132	4.383	−54	−66	−9
Cerebelum_4_5_L	132	4.191	−27	−36	−27
zALFF					
Calcarine_R	269	4.883	12	−72	9
Calcarine_L	269	4.467	−6	−90	6
ParaHippocampal_R	269	4.443	21	−39	−12
Temporal_Inf_L	80	4.661	−54	−66	−9
SmKCCReHo					
Calcarine_R	175	4.624	18	−66	6
SzKCCReHo					
Calcarine_R	165	4.649	18	−66	6
SmCoheReHo					
Calcarine_R	200	4.536	21	−66	6
Cuneus_R	200	4.025	6	−93	15
Calcarine_L	200	3.544	−9	−69	15
SzCoheReHo					
Calcarine_R	196	4.533	18	−69	6
Cuneus_R	196	4.065	6	−93	15
Calcarine_L	196	3.584	−9	−72	15

**Table 3:** Brain regions with decreased activity in athletes compared to novices.

Negative region label	Cluster extent	*t*-value	MNI coordinates		
			x	y	z
mfALFF					
Frontal_Mid_2_R	77	−4.241	48	36	27
zfALFF					
Frontal_Sup_2_R	61	−4.521	24	63	6
Frontal_Mid_2_R	82	−4.346	48	36	27
SmKCCReHo					
Frontal_Sup_2_R	326	−4.664	24	24	54
Frontal_Mid_2_R	326	−4.946	36	21	36
SzKCCReHo					
Frontal_Sup_2_R	200	−3.281	24	51	39
Frontal_Mid_2_R	200	−4.943	36	21	36

### 3.3 Regional homogeneity

**Figure 3:**
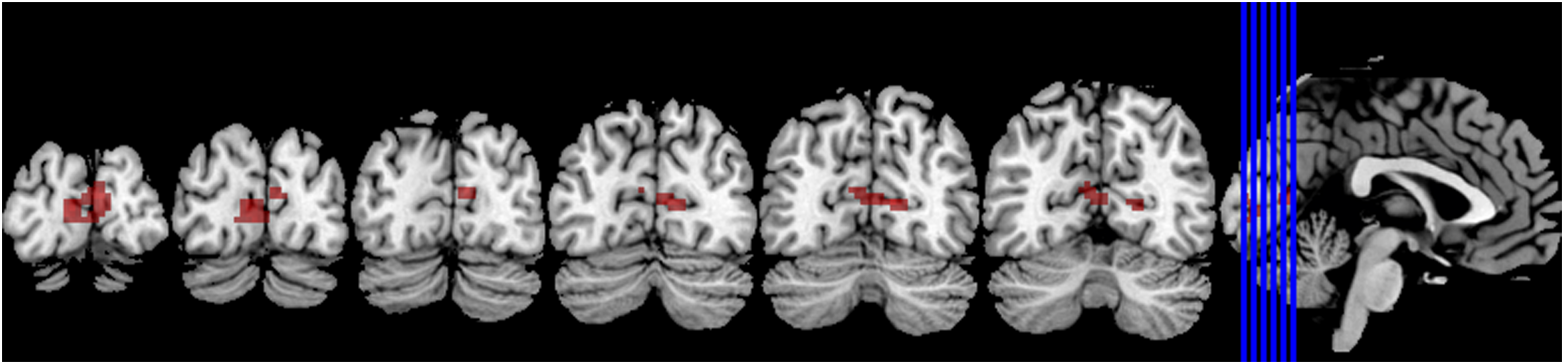
The brain regions primarily activated in athletes compared to normal individuals are located around the bilateral calcarine sulcus.

Compared to the novice group, the athlete group showed a significant increase in smkccReHo and szkccReHo of the right calcarine cortex (*p* < 0.005). In addition, the athlete group exhibited a significant decrease in smkccReHo and szkccReHo of the right superior frontal gyrus and right middle frontal gyrus (*p* < 0.005). The athlete group also showed a significant increase in smcoheReHo and szcoheReHo of the right calcarine cortex, right cuneus, and left calcarine cortex (*p* < 0.005). These findings are presented in Tables 2, 3, and Figure 3.

### 3.4 Functional connection

**Figure 4:**
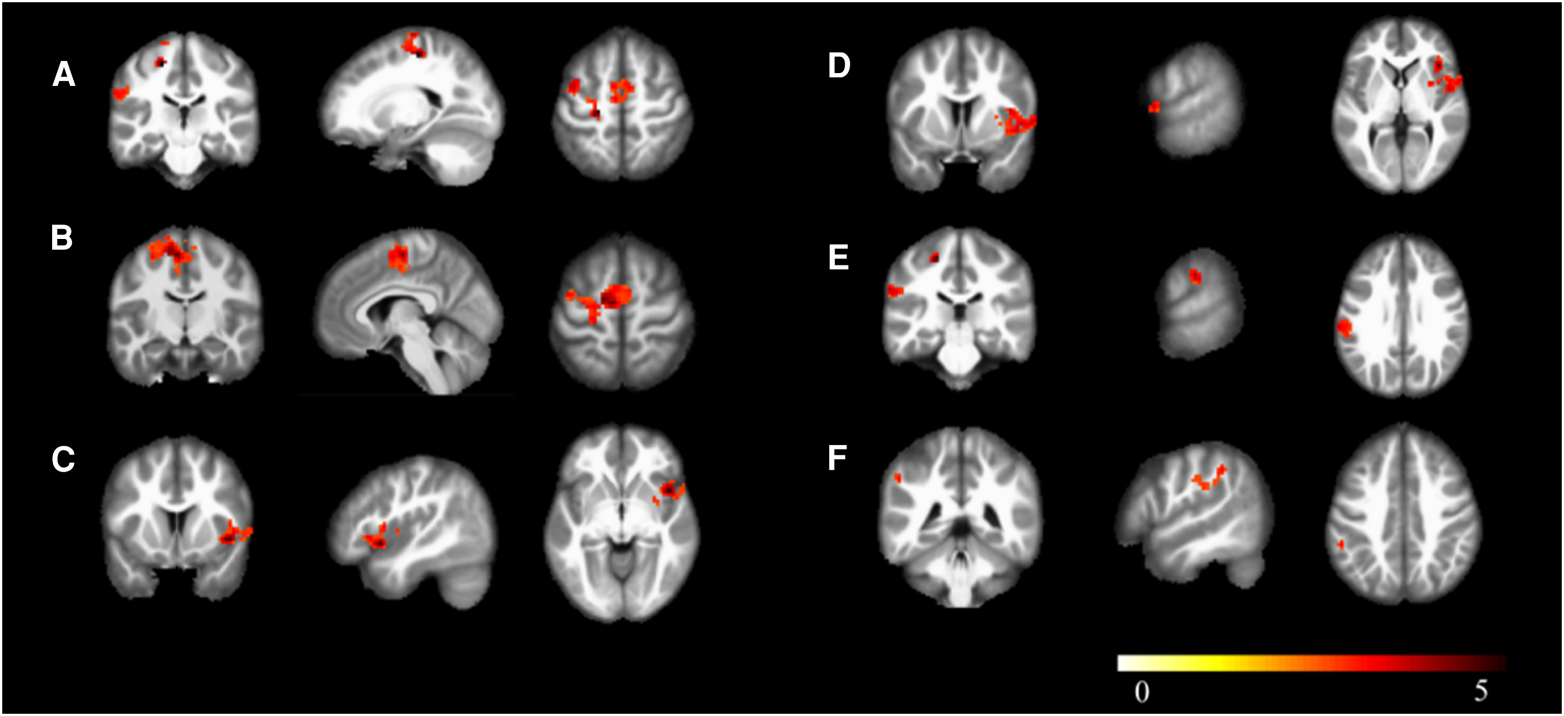
Brain regions with increased functional connectivity to the calcarine sulcus. (**A**) Precentral_L; (**B**) Supp_Motor_Area_L; (**C**) Insula_R; (**D**) Rolandic_Oper_R; (**E**) SupraMarginal_L; (**F**) Parietal_Inf_L; Cluster *p* < 0.05.

**Figure 5:**
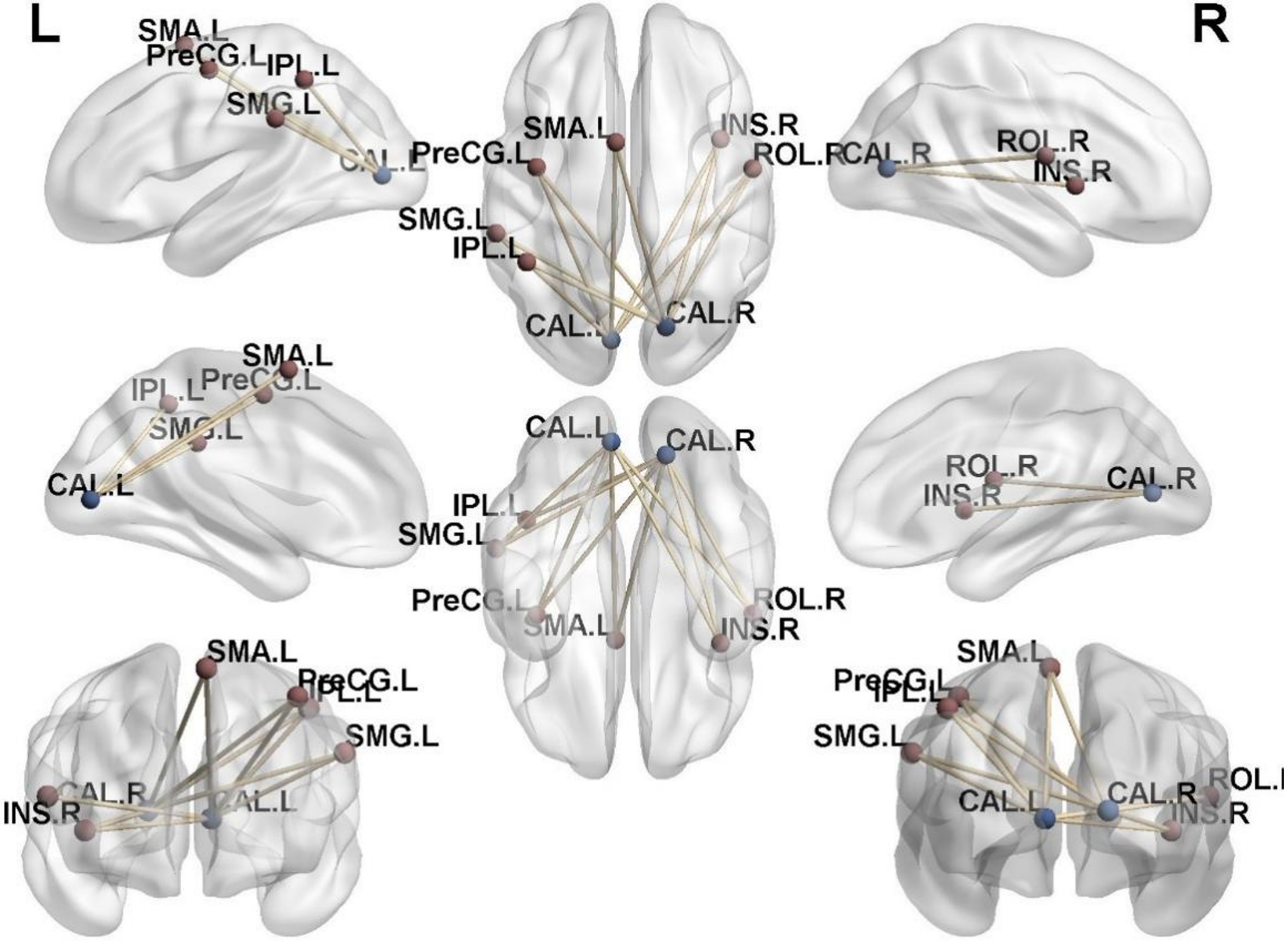
Functional connectivity structural diagram. Blue spheres representing the calcarine sulcus and red spheres representing other brain regions with increased functional connectivity to the calcarine sulcus.

In the athlete group compared to the novice group, there was an increased connectivity between Calcarine and several motor-related brain regions, including the Left Precentral Gyrus, Left Supplementary Motor Area, Left Precentral Gyrus, Right Insular Cortex, Right Rolandic Operculum, Left Supramarginal Gyrus, and Left Inferior Parietal Lobule (*p* < 0.01). These findings are presented in Table 4, Figures 4, 5.

**Table 4:** Brain regions with heightened functional connection in athletes compared to novices.

Positive region label	Cluster extent	*t*-value	MNI coordinates		
			x	y	z
FC					
Precentral_L	441	5.085	−21	−24	57
Supp_Motor_Area_L	441	4.312	−6	−12	63
Insula_R	252	4.580	45	12	−6
Rolandic_Oper_R	252	3.658	63	9	3
SupraMarginal_L	95	3.671	−66	−27	30
Parietal_Inf_L	95	3.150	−54	−42	42
zFC					
Precentral_L	375	4.989	−21	−24	57
Supp_Motor_Area_L	375	4.234	−6	−12	63
Insula_R	252	4.499	45	12	−6
Rolandic_Oper_R	252	3.678	63	9	3
SupraMarginal_L	95	3.659	−66	−27	30
Parietal_Inf_L	95	3.166	−54	−42	42

### 3.5 Correlation

We selected the calcarine cortex as the region of interest and extracted the ALFF and ReHo values of the calcarine cortex. Pearson correlation tests were performed between these imaging metrics and various behavioral scores, revealing no significant correlations between the behavioral and imaging data.

## 4 Discussion

This study found that the majority of differential brain areas between the group of volleyball players and the general population were concentrated in the visual system. Consistent results from metrics such as mALFF and zALFF showed involvement of the calcarine cortex, inferior temporal gyrus, and parahippocampal gyrus. These areas are associated with preliminary processing of external information, processing of visual features, object recognition and identification, perception of object and external spatial location. And the activation of the cerebellum is related to cognitive and sensory-motor functions. These findings are consistent with numerous behavioral studies by earlier researchers, demonstrating that after long-term specialized training, the brain plasticity of volleyball players undergoes beneficial changes in individuals (33, 34).

The results of ReHo further indicate the superior performance of volleyball players in visual information processing. The activity consistency in the bilateral primary visual cortex and the region surrounding the right cuneus was stronger in the athlete group, reflecting the enhanced visual processing ability in their brains compared to the general population. Furthermore, focusing on the differential brain areas in the bilateral primary visual cortex, we conducted whole-brain functional connectivity analysis at the voxel level. Our results further revealed that key connections in the brains of athletes, specifically between the bilateral primary visual cortex and the left primary somatosensory cortex and supplementary motor area, were significantly stronger compared to the novice group. Moreover, in terms of higher-level cognitive functions, the functional connectivity between the bilateral primary visual cortex and the right insula, left posterior cingulate cortex, and left precuneus was also significantly stronger in athletes compared to the novice group.

In a study involving 87 professional volleyball players and 67 non-athletes, it was found that athletes performed better in executive control tasks and visual-spatial attention processing tasks (17). Additionally, in sports, the most efficient or skilled actions are considered optimal (11). Athletes are able to predict the sensory outcomes of motor commands, integrate these predictions with actual sensory feedback, make judgments about their body and the external world based on this integration, and further adjust the sensorimotor feedback loop based on these judgments to optimize the cost and reward of movement and maximize performance (11). These findings align with our research results, which indicate enhanced local activity in the primary visual cortex and increased functional connectivity between the primary visual cortex and somatosensory cortex, as well as the supplementary motor cortex and other brain regions involved in visual information processing, cognitive control, decision-making, and visual-spatial processing in volleyball players (32). These changes optimize the brain's visual processing and body control during movement.

Although this study did not reveal statistically significant differences between the two groups in terms of auditory and visual reaction times, the correlation analysis between behavioral and imaging data did not show any statistical differences either. However, the differences between athletes and non-athletes have been well-established by other researchers (33). Previous studies have shown that athletes perform better in their own specific sports compared to non-athletes. However, it does not necessarily mean that they will demonstrate the same superiority when switching to different sports (11). This is similar to the findings of our experiment, which was an observational study. In our experiment, we tested the reaction times to sound and light using an audiovisual reaction device. This device was not part of the athletes' training, so they did not perform significantly better than the non-athlete group during the initial test. However, it is possible that the athlete group would show better performance after a period of training on the audiovisual reaction device. This would require further validation.

From the perspective of growth and development, there is a surge in synaptic generation in the visual areas before and after birth. Subsequently, the growth process primarily involves synaptic elimination, which relies on activity. Synapses are preserved in active brain cortex regions while gradually disappearing in inactive areas (35). Through this process of synaptic pruning, the visual system can gradually self-regulate based on environmental demands and sensory input. This implies that targeted training in the early stages can preserve a greater number of synapses in the brain cortex associated with the trained activity, providing a solid neurological foundation in the corresponding training domain.

Based on previous research, the mechanism of exercise in regulating brain neuroplasticity involves the action of various neurotrophins, such as monoamines, brain-derived neurotrophic factor (BDNF), and Insulin-Like Growth Factor 1(IGF-1) (19). Particularly, BDNF, as a primary brain plasticity modulator, exhibits heightened sensitivity to exercise. It not only regulates the central nervous system but also exerts certain control over the peripheral nervous system, with a more enduring impact compared to nerve growth factor (NGF), vascular endothelial growth factor (VEGF), and fibroblast growth factor 2 (FGF-2). In addition to changes in neurotransmitters, the focal point of exercise-induced neural plasticity lies in the intermediation of synaptic genesis and dendritic spine formation. Exercise enhances synaptic genesis and dendritic spine formation in multiple brain regions, especially the hippocampus and somatosensory cortex (36). Furthermore, exercise-induced synaptic-based long-term potentiation (LTP) is also considered a crucial aspect in influencing brain plasticity (37).

Some researchers have expressed doubts about the substantial benefits of early specialization in sports training for long-term youth development, and the underlying mechanisms remain unclear (38). However, this study focused on volleyball athletes and found that, in terms of brain plasticity, early specialization training contributes to increased activation in the visual cortex of young individuals. It also strengthens functional connections between the visual cortex and multiple sensory, motor, and cognitive-related cortices. This reflects enhanced brain function in visual-motor processing, attention-movement regulation, and improved neural efficiency in areas related to executive control. Other studies have also found adaptations in visual and motor-related processes due to long-term training, including modifications in neural functioning, gray matter, and white matter structures (34). This evident brain plasticity, along with the direct relationship between neural function and visual-motor response capabilities, offers significant potential for sports performance. And this unique ability of higher organisms to adapt to the environment by changing ourselves through learning and experience is specific to us. It is crucial in the process of evolution for our species (19). Furthermore, other researchers have discovered that adolescents participating in team sports exhibit higher levels of life satisfaction, positive emotions, and self-esteem (38). These findings provide further evidence for the cultivation of early sporting talent and broader youth development. In this study, we only focused on observing the effects of volleyball participation, a team sport, on brain plasticity among volleyball athletes, without further collecting social network data of the athletes (39). In future research, we will take this into consideration and further collect relevant data, which may help to address this limitation.

Furthermore, early researchers have found that the acquisition of individual skills not only affects brain activity but also leads to gray matter changes in many brain regions (11). In our subsequent research, we will further investigate the structural changes in the brains of volleyball athletes compared to ordinary university students. This will allow us to explore the brain's adaptations resulting from skill acquisition in competitive sports training. If there are neuroplastic changes induced by prior sports training, it suggests that the use-dependent plasticity induced by training can contribute to brain function remodeling when facing issues such as disabilities requiring movement rehabilitation (40). This highlights the beneficial role of early training in promoting the reconstruction of motor function following neural injuries.

## 5 Conclusion

This study demonstrates that long-term volleyball training significantly enhances the activity intensity of the visual cortex in the brain. It also notably strengthens the functional connectivity between the visual cortex and several other brain regions, including the primary somatosensory cortex, supplementary motor area, insula, superior parietal lobule, and inferior parietal lobule. These findings highlight superior visual-motor cognitive abilities among volleyball players, providing compelling evidence for the neuroplasticity of brain function in volleyball. Moreover, the study suggests that volleyball, as a team sport, contributes to the development of visual cognitive and motor functions, which offers valuable insights for the cultivation of early sports talents and broader youth development. Additionally, it provides new avenues for the prevention and treatment of motor function disorders.

## Data Availability

The raw data supporting the conclusions of this article will be made available by the authors, without undue reservation.
