# Supplementary material for: Sports promote brain evolution: a resting-state fMRI study of volleyball athlete
Source: Front Sports Act Living. 2024 May 2;6:1393988. doi: 10.3389/fspor.2024.1393988 (PMC11098564; doi:10.3389/fspor.2024.1393988)
Supplement: Supplementary file 1 [file Table1.docx]

**Table 1. Basic Information**

| Parameter | Athletes | Novices |
| --- | --- | --- |
|  | Mean±SD | Mean±SD |
| Age | 21.00 ± 1.30^a^ | 20.40 ± 0.70 |
| Years of sports experience | 10.30 ± 1.50 | 0.70 ± 0.21 |
| FMS | 16.8± 1.87 ^a^ | 17.50± 1.08 |
| Auditory reaction time | 174.22 ± 32.49 ^a^ | 184.04± 23.75 |
| Visual reaction time | 622.42 ±95.02 ^a^ | 591.85±57.14 |
| SDMT | 69.5 ±7.83 ^a^ | 68.80±9.28 |
| HAM-A | 5.40 ± 4.12 ^a^ | 7.00±4.64 |
| HAMD | 6.50 ± 5.08 ^a^ | 8.80 ± 5.65 |

Note：FMS, Functional Movement Screen; SDMT, Symbol digit substitution modalities test; HAM-A, The Hamilton Rating Scale for Anxiety; HAMD, Hamilton depression scale; ^a^ no significantly different to novices(P>0.05).

**Table 2. Brain regions with heightened activity in athletes compared to novices**

| Positive Region Label | | Cluster Extent | t-value | MNI Coordinates | | |
| --- | --- | --- | --- | --- | --- | --- |
|  |  |  |  | x | y | z |
| mALFF |  |  |  |  |  |  |
|  | ParaHippocampal_R | 221 | 5.021 | 21 | -39 | -12 |
|  | Calcarine_R | 221 | 4.522 | 12 | -72 | 9 |
|  | Calcarine_L | 221 | 4.099 | -6 | -87 | 6 |
|  | Temporal_Inf_L | 132 | 4.383 | -54 | -66 | -9 |
|  | Cerebelum_4_5_L | 132 | 4.191 | -27 | -36 | -27 |
| zALFF |  |  |  |  |  |  |
|  | Calcarine_R | 269 | 4.883 | 12 | -72 | 9 |
|  | Calcarine_L | 269 | 4.467 | -6 | -90 | 6 |
|  | ParaHippocampal_R | 269 | 4.443 | 21 | -39 | -12 |
|  | Temporal_Inf_L | 80 | 4.661 | -54 | -66 | -9 |
| SmKCCReHo |  |  |  |  |  |  |
|  | Calcarine_R | 175 | 4.624 | 18 | -66 | 6 |
| SzKCCReHo |  |  |  |  |  |  |
|  | Calcarine_R | 165 | 4.649 | 18 | -66 | 6 |
| SmCoheReHo |  |  |  |  |  |  |
|  | Calcarine_R | 200 | 4.536 | 21 | -66 | 6 |
|  | Cuneus_R | 200 | 4.025 | 6 | -93 | 15 |
|  | Calcarine_L | 200 | 3.544 | -9 | -69 | 15 |
| SzCoheReHo |  |  |  |  |  |  |
|  | Calcarine_R | 196 | 4.533 | 18 | -69 | 6 |
|  | Cuneus_R | 196 | 4.065 | 6 | -93 | 15 |
|  | Calcarine_L | 196 | 3.584 | -9 | -72 | 15 |

**Table 3. Brain regions with decreased activity in athletes compared to novices**

| Negative Region Label | | Cluster Extent | t-value | MNI Coordinates | | |
| --- | --- | --- | --- | --- | --- | --- |
|  |  |  |  | x | y | z |
| mfALFF |  |  |  |  |  |  |
|  | Frontal_Mid_2_R | 77 | -4.241 | 48 | 36 | 27 |
| zfALFF |  |  |  |  |  |  |
|  | Frontal_Sup_2_R | 61 | -4.521 | 24 | 63 | 6 |
|  | Frontal_Mid_2_R | 82 | -4.346 | 48 | 36 | 27 |
| SmKCCReHo |  |  |  |  |  |  |
|  | Frontal_Sup_2_R | 326 | -4.664 | 24 | 24 | 54 |
|  | Frontal_Mid_2_R | 326 | -4.946 | 36 | 21 | 36 |
| SzKCCReHo |  |  |  |  |  |  |
|  | Frontal_Sup_2_R | 200 | -3.281 | 24 | 51 | 39 |
|  | Frontal_Mid_2_R | 200 | -4.943 | 36 | 21 | 36 |

**Table 4. Brain regions with heightened functional connection in athletes compared to novices**

| Positive Region Label | | Cluster Extent | t-value | MNI Coordinates | | |
| --- | --- | --- | --- | --- | --- | --- |
|  |  |  |  | x | y | z |
| FC |  |  |  |  |  |  |
|  | Precentral_L | 441 | 5.085 | -21 | -24 | 57 |
|  | Supp_Motor_Area_L | 441 | 4.312 | -6 | -12 | 63 |
|  | Insula_R | 252 | 4.580 | 45 | 12 | -6 |
|  | Rolandic_Oper_R | 252 | 3.658 | 63 | 9 | 3 |
|  | SupraMarginal_L | 95 | 3.671 | -66 | -27 | 30 |
|  | Parietal_Inf_L | 95 | 3.150 | -54 | -42 | 42 |
| zFC |  |  |  |  |  |  |
|  | Precentral_L | 375 | 4.989 | -21 | -24 | 57 |
|  | Supp_Motor_Area_L | 375 | 4.234 | -6 | -12 | 63 |
|  | Insula_R | 252 | 4.499 | 45 | 12 | -6 |
|  | Rolandic_Oper_R | 252 | 3.678 | 63 | 9 | 3 |
|  | SupraMarginal_L | 95 | 3.659 | -66 | -27 | 30 |
|  | Parietal_Inf_L | 95 | 3.166 | -54 | -42 | 42 |
